# Electrochemical Methods for the Analysis of Trace Tin Concentrations—Review

**DOI:** 10.3390/ma16247545

**Published:** 2023-12-07

**Authors:** Malgorzata Grabarczyk, Edyta Wlazlowska, Marzena Fialek

**Affiliations:** Department of Analytical Chemistry, Institute of Chemical Sciences, Faculty of Chemistry, Maria Curie-Sklodowska University, 20-031 Lublin, Poland; malgorzata.grabarczyk@mail.umcs.pl (M.G.); marzena.fialek@mail.umc.pl (M.F.)

**Keywords:** tin, electrochemical methods, stripping voltammetry, polarography, potentiometry

## Abstract

Tin determination allows for the monitoring of pollution and assessment of the impact of human activities on the environment. The determination of tin in the environment is crucial for the protection of human health and ecosystems, and for maintaining sustainability. Tin can be released into the environment from various sources, such as industry, transportation, and electronic waste. The concentration of tin in the environment can be determined by different analytical methods, depending on the form of tin present and the purpose of the analysis. The choice of an appropriate method depends on the type of sample, concentration levels, and the available instrumentation. In this paper, we have carried out a literature review of electrochemical methods for the determination of tin. Electrochemical methods of analysis such as polarography, voltammetry, and potentiometry can be used for the determination of tin in various environmental samples, as well as in metal alloys. The detection limits and linearity ranges obtained for the determination of tin by different electrochemical techniques are collected and presented. The influence of the choice of base electrolyte and working electrode on signals is also presented. Practical applications of the developed tin determination methods in analyzing real samples are also summarized.

## 1. Introduction

Tin is a shiny silvery metal in group 14 of the periodic table. It is well known for its historical importance, versatility, and many applications. Its addition into copper alloys to make bronze marked a major technological achievement in human history. In ancient times, tin was also used to make mirrors and decorative objects. Its ductility, low melting point, and corrosion resistance make it a valuable material for a wide range of industrial and technological applications. Tin compounds are readily used as catalysts and stabilizers in the plastics industry. Nowadays, tin is used to coat other metals because it forms a thin anti-corrosion layer that protects objects from rust. This type of protection is mainly used in the food industry for cans or steel trays. It is also used in the electronics industry for touch screens and solar cells [1,2,3,4,5].

Tin occurs naturally in small amounts in soils and sediments. The concentration of tin in soils can vary depending on the geological characteristics of the region. Soil concentrations typically range between 1–4 ppm, sometimes falling below 0.1 ppm, while peat can be as high as 300 ppm. Although tin occurs naturally in the environment, human activities can significantly affect its distribution and concentration, potentially leading to environmental problems and the need for appropriate management and remediation in areas where tin mining and processing take place. The toxicity of most tin compounds is relatively low and they are not considered to be very hazardous. However, as with many chemicals, exposure to high concentrations or chronic exposure to certain tin compounds can cause adverse health effects. Organic tin compounds are more toxic than inorganic ones. This is due to their poor absorption and relative insolubility in inorganic tin compounds. Organic tin compounds, on the other hand, are better absorbed and easily penetrate between human organs. The exposure limit value for tin and its inorganic compounds has been set at 2 mg m^−3^. The WHO has set the maximum tolerable level of tin in food at 250 µg g^−1^. The levels of tin in environmental and biological samples cover a wide range, from µg g^−1^ in canned foods to ng g^−1^ and even pg g^−1^ in water. Consumption of food containing inorganic tin at a concentration of 200 mg kg^−1^ may lead to side effects. Too much tin in the body can affect the functioning of the nervous system, cause thymus problems, and weaken the immune system. A deficiency, on the other hand, manifests itself in stunted hair and nail growth, vision problems, and delayed reaction time to stimuli. It is, therefore, important to balance the concentration of tin in the human body [1,2,4,5,6,7,8].

The interest in the determination of tin is related to its wide application in various fields. Many methods for the determination of tin have been described in the literature. Frequently used methods for the determination of tin include atomic absorption spectroscopy [9], spectrophotometry [10,11,12], atomic emission spectroscopy [13], inductively coupled plasma-mass spectrometry (ICP–MS) [14], inductively coupled plasma-optical emission spectroscopy (ICP–OES) [15], X-Ray fluorescence spectrometry [16], liquid–solid chromatography [17], and chemiluminescence [18]. Although many analytical techniques, such as atomic spectroscopy with high trace element detection capabilities, have been used for many years, their high cost and, in some cases, low sensitivity and selectivity mean that there is a need for more sensitive and cheaper methods. Of these, ICP–MS and ICP–OES only allow the determination of organic and total tin concentrations. These methods are expensive and do not allow the selective determination of Sn(II) and Sn(IV) ions [9,10,11,12,13,14,15,16,17,18].

Electrochemical methods can be an interesting alternative to other methods for the determination of traces of tin. Electrochemical methods rely on the measurement of electrical quantities such as potential, current, or charge. The main advantages of electrochemical methods over other detection systems are that high sensitivities, wide ranges of linearity, and low instrument costs are achieved [19]. Electroanalytical methods for the detection of tin include stripping voltammetry [20,21,22,23,24,25,26,27,28,29,30,31,32,33,34,35,36,37,38,39,40,41,42,43,44,45,46], polarography [47,48,49,50,51,52,53,54,55,56,57], potentiometry [58,59,60], and coulometry [61]. In the literature, most of the works on the electrochemical determination of tin are devoted to voltammetric methods. In stripping voltammetry methods, measurement is carried out in two stages. In the first stage, called concentration, a labeled component accumulates on the electrode; in the second stage, called stripping, the accumulated component undergoes an electrode reaction as a result of the change in the potential of the working electrode. In the stripping stage, the voltammetric curve is recorded as a peak. There are three known methods of stripping voltammetry, which differ in the way the analyte is accumulated on the working electrode. If the accumulation is by adsorption, the method is called adsorptive stripping voltammetry (AdSV). In this method, during the stripping stage, the determined substance is oxidized or reduced depending on the potential applied to the working electrode; in a few cases, the signal is obtained based on a desorption process. In anodic stripping voltammetry (ASV), the first step involves an electrolytic process carried out by electrochemical reduction of the labeled substance at a constant potential, which, depending on the nature of the separated metal and the electrode material, dissolves or forms a film on the electrode surface. After electrolytic concentration, the electrode is polarized in the anodic direction, and at an appropriate potential, the labelled metal is oxidized. Anodic stripping voltammetry is the oldest stripping method in use. In contrast, cathodic stripping voltammetry (CSV) involves an electrode reaction in the first step, resulting in the formation of a poorly soluble compound on the surface of the working electrode. In the stripping step in this method, decreasing potential is applied to the working electrode. At this potential, the labelled metal is reduced [62,63,64]. The voltammetric methods developed for the determination of tin are summarized in Table 1. As you can see, the most frequently used method in the voltammetric analysis of tin is the ASV method, which uses different working electrodes and supporting electrolytes [20,21,22,23,24,25,26,27,28]. The lowest limit of detection for tin was obtained using the AdSV method. Due to the accumulation of tin with the complexing agent on the working electrode, higher sensitivity is achieved compared to the ASV method, allowing the detection of lower analyte concentrations. Only one paper on the determination of tin by cathodic stripping voltammetry has been published in the literature [33].

Polarography is an older technique than voltammetry. Polarography was introduced by Jaroslav Heyrovský in the 1920s. This electrochemical technique focuses on the study of redox reactions and is widely used in analytical chemistry. Polarographic methods have also been used for the determination of tin. The main difference between classical electrolysis and the polarographic method is that in the former, the total mass of the substances separated at the electrodes is determined, whereas in polarography, changes in the current flowing through the solution under test during electrolysis are studied. Polarographic methods should use working electrodes that are liquid electrodes with a continuously or periodically renewed surface, whereas voltammetric methods use working electrodes where the electrode area is constant [63,65,66]. Table 2 collects the works on the determination of tin by polarographic methods.

Tin(II) analysis is also carried out using potentiometric membrane sensors, known as ion-selective electrodes (ISEs) [58,59,60]. The main advantage of the ISE is that it provides precise analytical information. In this method, the electrode reaction takes place without an applied external voltage. The basis of potentiometry is the measurement of the electromotive force. The measurement involves examining the difference in electrochemical potential between the electrodes immersed in the diluent to be analyzed. ISE electrodes are provided with an ion-selective membrane, which separates the corresponding half–cell from the test solution. Such a membrane is sensitive to the activity of a particular type of ion. The main advantage of the ISE is that it provides a rapid yet accurate determination of the analyte [67,68,69]. Only a few ion-selective electrodes have been proposed for the determination of tin(II). Table 3 shows information on the composition of the electrode membranes and the detection limits obtained.

This paper reviews the works described in the literature on electroanalytical methods for the determination of tin, with particular reference to the basic electrolytes, complexing agents used, types of working electrodes, detection limits obtained, and the possibility of applying the methods to the determination of real samples.

## 2. Types of Techniques Used

### 2.1. Polarography

Ten papers related to the determination of tin by polarographic methods were found. Table 2 collects information on the detection limits, linearity ranges obtained, working electrodes used, and interferences investigated that may affect the tin signal. Real samples in which tin was determined by the developed methods are also presented.

**Figure 1:**
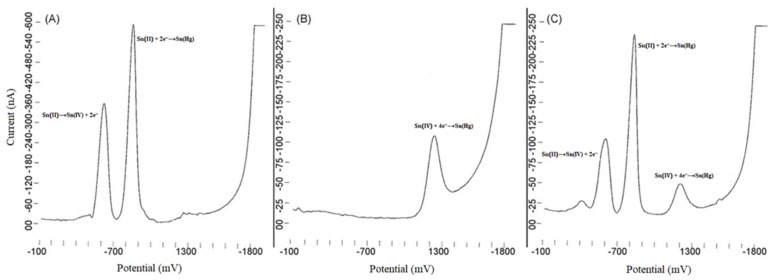
Polarograms of tin species solutions in carbonate/bicarbonate solution (0.2 mol L^−1^) and malonate ions (0.5 mol L^−1^). (**A**) 30 µg mL^−1^ Sn(II), (**B**) 30 µg mL^−1^ Sn(IV), and (**C**) 10 µg mL^−1^ Sn(II) and Sn(IV). After re-elaboration from Ref. [49] with permission from the publisher.

As can be seen by analyzing Table 2, the most frequently used polarographic method is differential pulse polarography (DPV). In DPV methods, the current is sampled at two different points in the waveform, resulting in a differential output signal. This makes the developed methods highly sensitive. Weber proposed a DPV method for the determination of tin using the DME as the working electrode. The optimized procedure allowed for the determination of tin at the lowest concentration of 8.4 × 10^−10^ mol L^−1^, the lowest detection limit reported in the literature, using the polarography technique. Another advantage of the developed method is the achievement of a wide range of linearity in the determination of tin, from 8.4 × 10^−9^ to 4.2 × 10^−5^ mol L^−1^ [50]. Of course, there are also works in which the detection limit obtained is less satisfactory, being 5 orders of magnitude higher. This results in a significant reduction in the sensitivity of tin determination. Figure 1 shows the polarograms obtained for the tin solutions [49].

### 2.2. Voltammetry

Among the electrochemical methods for the determination of tin, stripping voltammetry offers many advantages, such as high sensitivity, accuracy, simplicity of the proposed analytical procedures, low cost of instrumentation and reagents, but also a wide range of applications of the developed procedures [19].

**Figure 2:**
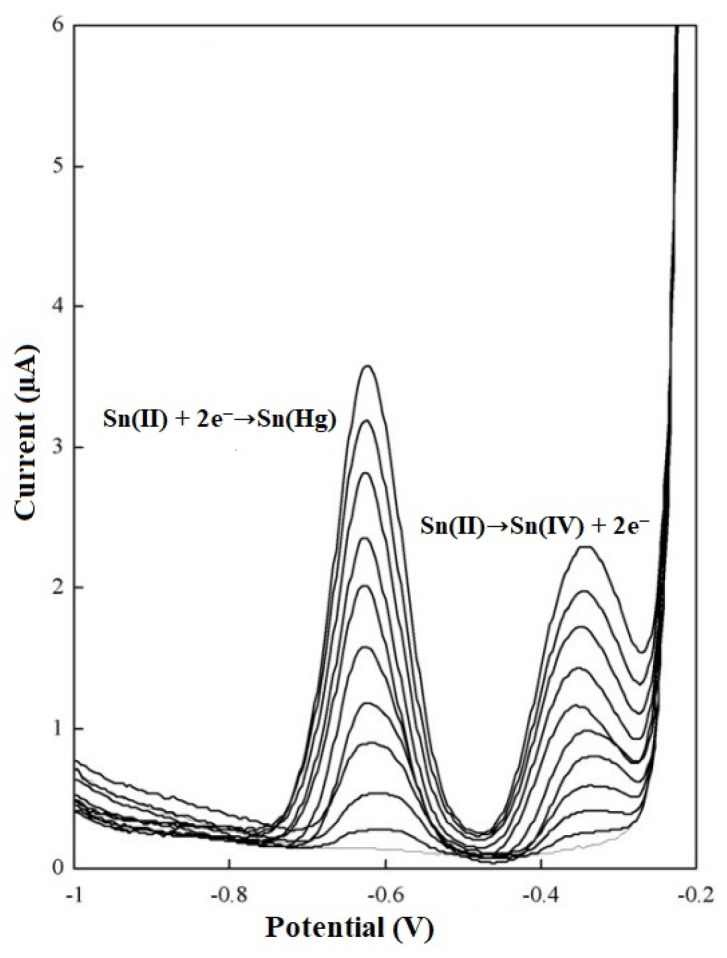
Stripping voltammograms for 10 successive additions of 10 µg L^−1^ tin(II) obtained at BiFE. Supporting electrolyte 0.1 mol L^−1^ acetate buffer, 10 mg L^−1^ Bi, and 150 µmol L^−1^ catechol. Deposition at −1.0 V for 60 s. After re-elaboration from Ref. [26] with permission from the publisher.

Table 1 summarizes the developed methods for the determination of tin by stripping voltammetry, including information on working electrodes used, complexing agents, linearity ranges obtained, limits of detection, interferences studied, and real samples on which the developed methods were used to determine tin. The use of a buildup and stripping step in the procedures significantly increased the sensitivity of the determination of tin, making it possible to determine very low concentrations. The most commonly used electrode in voltammetric methods for the determination of tin has been the hanging mercury electrode, due to its high sensitivity, repeatability, and accuracy. However, due to the toxicity of mercury, other electrodes were introduced to eliminate or reduce the use of this metal in electrode manufacture. A bismuth film electrode was introduced for the determination of tin by anodic stripping voltammetry. Examples of voltammograms recorded during the determination of tin using a bismuth electrode are shown in Figure 2 [26]. Unfortunately, the sensitivity of the method decreased, and the detection limits obtained were not as low as when using a mercury-based electrode. Another electrode that replaced the mercury-based electrode was the carbon paste electrode. By using this electrode and a complexing agent, which was BPR, a sensitive and selective method for the determination of traces of tin was achieved [24]. In this case, the detection limit was of the same order of magnitude as the detection limit obtained with a hanging drop mercury electrode. The detection limit can also be lowered by increasing the accumulation time of the element being determined on the surface of the working electrode. Very low concentrations of tin required long accumulation times to build up sufficient material on the electrode for accurate measurement. In the paper [35], the accumulation time was 600 s and thus, the lowest detection limit was obtained. The authors in [39] calculated detection limits at two different accumulation times. Increasing the accumulation time of the tin–oxine complex on the HMDE electrode by up to 240 s resulted in a detection limit that was one order of magnitude lower than that obtained, using a 60 s accumulation time [39]. In conclusion, the introduction of an accumulation and stripping step results in a significant reduction in the detection limit and a high increase in sensitivity, accuracy, and selectivity of the developed tin determination methods.

### 2.3. Potentiometry

A unique feature of potentiometry is that it measures the concentration of free ions and not the total ion concentration. Therefore, the potentiometric methods described determine the concentration of Sn(II) ions [58,59,60]. Three ion-selective electrodes have been proposed for the determination of stannous. Table 3 describes selected characteristics of the ion-selective electrode and the developed method for the determination of tin. The lowest detection limit obtained in the determination of tin with the ion-selective electrode was 8.0 × 10^−7^ mol L^−1^ [58]. Potentiometric techniques used for the determination of Sn(II) are characterized by a wide range of linearity. The range of linearity obtained in the work [58] was in the range from 5.0 × 10^−7^ mol L^−1^ to 1.0 × 10^−1^ mol L^−1^. This was the widest range of linearity among the methods discussed in that article [58]. For ion-selective electrodes, dynamic response time is an important factor. The ion-selective electrodes developed for the determination of stannous are characterized by a short response time, with the electrodes reaching equilibrium in the range of 15 to 20 s. The lifetimes of the electrodes, during which the results obtained for the determination of tin were reproducible, were also investigated. The longest lifetime was up to 90 days, and it was obtained for an electrode in which the ionophore was DB18C6 [59].

## 3. Measurement Conditions

### 3.1. Working Electrodes

Working electrodes play a key role in the electrochemical analysis of substances. They are used to measure current response as a function of applied potential or electrochemical processes, as well as to determine the concentration of analytes in the solution. Ideally, the electrode should have a high signal-to-noise ratio. Mercury electrodes are the most commonly used electrodes in voltammetry methods. They have a smooth surface, excellent repeatability, polarizability, and a wide potential range. Unfortunately, their use creates the possibility of mercury compounds being released into the environment. Therefore, new solutions are constantly being sought to eliminate mercury-based electrodes [70,71]. Mercury electrodes used for trace tin determination include static mercury drop electrode (SMDE) [47,48,49], dropping mercury electrode (DME) [50,51,52], hanging mercury drop electrode (HMDE) [20,21,22,23,34,35,36,37,38,39,51,52], and mercury film electrode (MFE) [40,41]. As you can see, the determination of tin by adsorptive stripping voltammetry has most commonly been carried out using HMDE as the working electrode. The use of this electrode has resulted in the lowest detection limit of trace tin methods developed to date. In order to obtain a low detection limit, van den Berg et al. proposed the accumulation of a tin–tropolone coupling on the HMDE electrode. The voltammogram showed two peaks formed by the reduction of Sn(IV) to Sn(II) and then to Sn(0). The proximity of the oxidation and reduction peak potentials showed that the redox process is reversible. The difference between the potentials of the Sn(IV)/Sn(II) stages was small, since tin is present in both oxidation stages as adsorbed complexes [35]. A replacement for the HMDE electrode was proposed by Adeloju and Pablo, and involved using a glassy carbon electrode, over which a layer of mercury was deposited for the determination of tin.

As liquid electrodes are used in polarographic methods, the DME has been the most commonly used electrode for the determination of tin. This electrode consists of a small droplet of mercury at the end of a capillary tube, with size and frequency precisely controlled. When the mercury droplet comes into contact with the solution, redox reactions occur on the surface of the electrode. Depending on the purpose of the experiment, different substances can be tested, and the electrode potential can be precisely controlled. As a result of the redox reactions taking place in the DME, a current is generated. When this current is measured, it can provide information about the concentration of electroactive substances in the solution. The DME is particularly valuable because of its reproducibility, reliability, and ability to control the electrode surface. These characteristics make the DME well-suited for studying the kinetics and thermodynamics of various electrochemical reactions. The use of this electrode in the work [49] resulted in a very low tin detection limit of 8.42 × 10^−10^ mol L^−1^. Unfortunately, the use of this electrode produces a mercury drop with each measurement, which discourages the use of this method for the determination of tin.

The need to eliminate mercury electrodes in voltammetric techniques led to the development of new electrochemical sensors due to the toxicity of mercury and the need to dispose of it so that it does not enter the environment. The less toxic electrode used in tin determination techniques was the bismuth film electrode (BiFE). When using the BiFE, measurements were made by anodic stripping voltammetry from non-oxidized solutions, which reduced the analysis time. Simultaneous accumulation of tin and bismuth ions occurred on the working electrode, which was often a glassy carbon electrode. For this purpose, bismuth ions were added directly to the solution to be analyzed [23,24,25,26,27]. The paper [27] proposed to modify the surface of a bismuth electrode by introducing a mediator onto the electrode surface. The chosen mediator was zinc. It was deposited simultaneously with bismuth on the electrode surface. The determination of tin was carried out by anodic stripping voltammetry. The preparation of this electrode consisted of optimizing the concentration of Zn(II) and Bi(III) ions, as well as parameters such as potential and ion accumulation time on the working electrode. The resulting linearity range and detection limit using the zinc–mediated BiFE confirmed increased sensitivity for tin determination compared to unmodified BiFE electrodes [27]. A novel electrode solution was the introduction of a solid bismuth microelectrode for voltammetric measurements. When using these electrodes, it is not necessary to introduce bismuth ions into the voltammetric cell, as in the case of film bismuth electrodes, where it is necessary to form a film on the electrode surface. This advantage simplifies the analysis and reduces the measurement time. A solid bismuth microelectrode was used for the determination of tin by adsorptive stripping voltammetry. To ensure reproducible measurements and a well-shaped signal, the electrode was activated by applying a potential of −2.5 V to the electrode for 5 s prior to the tin accumulation step, with the complexing agent on the electrode. The use of a solid microelectrode and cupferron as a complexing agent allowed a low detection limit of 2.1 × 10^−9^ mol L^−1^ to be obtained [42].

Prior et al. proposed a bismuth film electrode (BiFE) for the determination of tin by anodic stripping voltammetry. To support the use of the BiFE electrode as a working electrode, comparative studies were carried out with a mercury film electrode (MFE). Tests were conducted with a solution containing 2 mol L^−1^ sodium bromide, and calibration curves were plotted for tin concentrations ranging from 25 to 150 mg L^−1^. The calibration curves obtained allowed for the determination of the regression coefficient (R^2^) and the relative standard deviation (%RSD). Measurements with the BiFE were carried out from a nondeoxygenated solution, while MFE electrode measurements were carried out from deoxygenated and nondeoxygenated solutions. The %RSD results indicate that comparable performance can be obtained for bismuth electrodes in nondeoxygenated solutions and mercury film electrodes in deoxygenated solutions, as the relative standard deviation is 4.7% for the BiFE electrode and 4.0% for the MFE electrode under deoxygenated conditions. In contrast, the reproducibility of tin under nondeoxygenated conditions using the MFE results in a decrease of the tin peak height, with the %RSD as high as 19.3%. The deoxygenation time of the solution was 5 min, which significantly increased the analysis time. Comparing the peaks obtained with these electrodes, it can be seen that the peaks obtained with the BiFE are broader and worse shaped than the peaks with the MFE, whereas the peak height obtained with the bismuth film electrode is approximately twice as low as that of the mercury electrode. Despite this, the bismuth film electrode was used for further tin determination studies [25].

**Figure 3:**
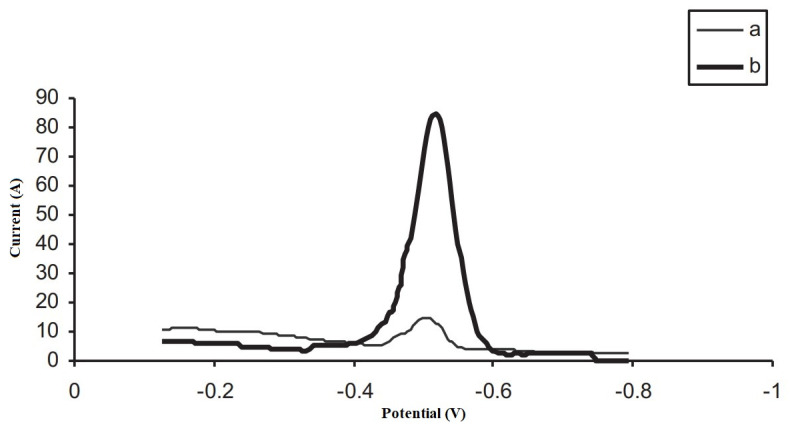
Voltammograms of 50 ng mL^−1^ of tin and borate buffer (pH 7.5). (a) Multiwalled carbon nanotube electrode, (b) cupferron-modified multiwalled carbon nanotube electrode. After re-elaboration from Ref. [31] and permission from the publisher.

Sobhanardakani et al. set out to develop a method for the determination of tin by anodic stripping voltammetry using a cupferron-modified multi-walled carbon nanotube electrode as the working electrode. Figure 3 shows the voltammograms recorded with an unmodified and a cupferron-modified electrode. As can be seen, the sensitivity of the tin determination increases significantly with the cupferron-modified electrode. Multiwalled carbon nanotube electrodes have excellent electroanalytical properties, such as a wide potential range, low background current, high assay sensitivity, and low detection limits. In this case, the detection limit for tin was 1.0 × 10^−9^ mol L^−1^ [31].

Another proposal to eliminate mercury electrodes in tin determination was to use a chemically modified glassy carbon electrode (CME). A method for the determination of Sn(IV) by cathodic stripping voltammetry using a glassy carbon electrode modified with Nafion^®^ (Aldrich Chemicals, St. Louis, MO, USA) has been reported. The recorded voltammograms for the glassy carbon electrode showed no peak for tin, whereas the use of the CME electrode showed two peaks for tin [33].

### 3.2. Supporting Electrolyte and Complexing Agent

The supporting electrolyte plays a key role in electroanalytical techniques such as polarography, voltammetry, and ion-selective electrode measurements. In order to develop an analyte determination procedure for electrochemical methods, it is very important to study the influence of electrolytes and, in some cases, additionally, complexing agents used in the procedures. Suitable electrolyte compositions for electroanalytical methods result in low detection limits and wide ranges of linearity for the method. The most commonly used supporting electrolytes in electrochemical methods have been acetate buffer [24,26,28,29,34,36,38,39,40,41,42], borate buffer [31], formate buffer [37], hydrochloric acid [30,32,48,55,56,57], oxalic acid [51], sodium bromide [25], and sodium chloride [33]. For voltammetric methods, acetate buffer was the most commonly used supporting electrolyte [24,26,28,29,34,36,38,39,40,41,42,43]. Its concentration in the solution from which the determination of tin was carried out was most often 0.1 mol L^−1^. In [41], the concentration of acetate buffer was reduced to 0.01 mol L^−1^. Using the acetate buffer, the pH of the solutions from which the measurements were made was in the range of 4.0 to 5.0. In polarographic methods, on the other hand, the primary electrolyte was usually a mixture of several components. The concentration of hydrochloric acid in the voltammetric methods for the determination of tin was 1.0 mol L^−1^. In the case of polarographic methods, hydrochloric acid was used in a concentration range from 0.1 mol L^−1^ to 3.5 mol L^−1^ [48,55,56,57].

In adsorptive voltammetric determinations, complexing agents are added to the base electrolyte and they play an important role in increasing the selectivity and sensitivity of the analysis by forming complexes with the tin to be determined. In the tin methods developed using adsorptive stripping voltammetry, the most commonly used complexing agents have been caffeic acid [28,29], tropolone [34,35], and catechol [36,40]. Other complexing agents have also been used, such as bromopyrogallol red (BPR) [24], 3,4-dihydroxybenzoic acid (DHBA) [37], 2,2-hydroxyphenylbenzoxazole (HBO) [38], 8-hydroxyquinoline (oxine) [39], and cupferron [42,43]. The highest concentration of complexing agent used was 1.73 × 10^−3^ mol L^−1^ [28,29]. The most commonly used concentration of complexing agent was n × 10^−5^ mol L^−1^ (where n ranged from 1 to 8) [24,35,37,38,41]. In the work [34], the complexing agent used was tropolone, and in this procedure, the lowest concentration of complexing agent used for the determination of tin was 4.0 × 10^−6^ mol L^−1^.

In the case of polarographic methods, both the base electrolyte and the complexing agent also play a key role. Qiong et al. proposed a method for the determination of tin by single sweep polarography. In this case, tin was determined from a solution containing oxalic acid and methylene blue (R^+^). Tin was shown to have a stable and well-defined cathodic peak in the presence of oxalic acid and methylene blue. It was also demonstrated that the addition of methylene blue significantly increased the height of the tin peak. The polarographic peak resulted from the reduction of tin(IV) to tin(II) at a potential of −0.30 V and further reduction of tin(II) to tin(0) [51]. Based on the results and observations, the following electrode reaction mechanism was established:

(1) $$R^{+}-Sn\left(IV\right)\Leftrightarrow R^{+}{Sn(IV)}_{ads}$$

(2) $$R^{+}-{Sn(IV)}_{ads}+2e\Leftrightarrow R^{+}Sn\left(II\right)$$

(3) $$R^{+}-Sn\left(II\right)\Leftrightarrow R^{+}{Sn\left(II\right)}_{ads}$$

(4) $$R^{+}-{Sn\left(II\right)}_{ads}+2e\Leftrightarrow R^{+}Sn$$

Another solution from which both Sn(II) and Sn(IV) could be determined was one containing a malonate ion. The use of such a chelating agent allowed the signals to be distinguished from the tin ions in the recorded polarogram. Carbonate ions were required in the solution to achieve sufficient differentiation, making the complexes very stable. In the absence of carbonate ions, the stability of the tin complexes was very low; on the other hand, if there were no malonate ions in the solution, the results obtained were completely different, and no selective determination of the tin and stannous ions was possible. It was also found that in a saline environment, both malonate and carbonate ions are involved in the chelation process, and therefore, the tin forms can be distinguished. For the stannous ion, two waves are assigned, corresponding to the oxidation and reduction reactions according to Equations (5) and (6). In the case of the stannous ion, there was a reduction according to Equation (7) [46].

(5) $${Sn}^{2+}⟶{Sn}^{4+}+{2e}^{-}$$

(6) $${Sn}^{2+}+{2e}^{-}⟶Sn(Hg)$$

(7) $${Sn}^{4+}+{4e}^{-}⟶Sn(Hg)$$

Pérez-Herranz et al. proposed a method for the determination of tin(II) in the presence of tin(IV) using differential pulse polarography. The effect of citric acid and hydrochloric acid concentrations on the determination of tin was studied. Total tin was determined in a solution of 0.1 mol L^−1^ hydrochloric acid since there was no complexing agent in the solution, and possible changes in the degree of oxidation did not affect the height of the peak and the potential at which it appeared. For the determination of Sn(II) in the presence of stannous(IV), the supporting electrolyte in which the tests were carried out consisted of 0.1 mol L^−1^ hydrochloric acid and 0.2 mol L^−1^ citric acid. It was possible to determine Sn(II) due to the fact that the peak of Sn(II) was well-formed, while no signal of Sn(IV) was recorded on the polarograph [55].

### 3.3. Detection Limits

The detection limit refers to the lowest concentration level or amount of a substance that can be accurately detected and measured by a particular electrochemical technique. Electrochemical methods can detect tin at very low concentrations, which is important in many fields, such as chemical analysis, quality control, and environmental testing. The detection limit is influenced by factors such as the choice of the working electrode, the supporting electrolyte, the pH of the solution in which the tests are carried out, and the choice of complexing agent in the case of adsorptive stripping voltammetry methods, but also the choice of analytical parameters, which can affect the signal obtained. Optimization of the analytical procedure involves selecting the best possible conditions to obtain low detection limits.

From Table 1, Table 2 and Table 3, it can be concluded that the most commonly used methods for the determination of tin are the stripping voltammetric methods, which give the lowest detection limits. For the methods discussed in this article, the lowest detection limit was obtained using adsorptive stripping voltammetry. A hanging drop mercury electrode was used as the working electrode and the detection limit obtained was 5.0 × 10^−12^ mol L^−1^. The highest detection limit for tin was 1.3 × 10^−5^ mol L^−1^. The technique used in this case for the determination of tin was differential pulse polarography.

The developed ion-selective electrodes for the determination of tin by potentiometric methods allowed for obtaining sufficient sensitivity with detection limits ranging from 4.0 × 10^−6^ to 8.0 × 10^−7^. The proposed ion-selective electrodes, in addition to their ability to obtain low detection limits, allowed fast response times; hence, these methods provided rapid analysis of tin samples [58,59,60].

Competing analytical methods for the determination of tin include atomic absorption spectrometry, inductively coupled plasma optical emission spectrometry (ICP–OES), and inductively coupled plasma mass spectrometry (ICP–MS). Comparing the detection limits for tin obtained by these methods with those obtained by voltammetry, it can be seen that voltammetry often gives lower or equal values for the detection limits obtained. The limits of detection obtained by atomic absorption spectrometry in the work [9] were 2.9 × 10^−8^ mol L^−1^, while in the paper [14], the use of the ICP–MS method allowed for the detection of tin at a concentration of 1.4 × 10^−9^ mol L^−1^. These results show that the use of spectroscopic methods for the determination of tin gives detection limits of the same order of magnitude as electrochemical methods, but spectroscopic methods use expensive equipment that not every laboratory is equipped with. Of course, it is possible to detect low concentrations of tin of 7.9 × 10^−10^ mol L^−1^ using the ICP–OES method for the determination of tin, but this method is more complicated than the techniques discussed in this article [15].

## 4. Influence of Foreign Ions and Organic Matrix

An important element in checking the analytical capability of the determination method used is to see what effect interferences present in the solution being analyzed have, the potential presence of which is highly probable when dealing with real samples. For the tin determination procedures described, the effect of the following metal ions co-present in the solution was checked: Na(I), K(I), Li(I), Ag(I), Cs(I), Ca(II), Mg(II), Al(III), Ni(II), Zn(II), Ba(II), Co(II), Hg(II), Fe(II), Mn(II), Cd(II), La(III), Ti(III), Ga(III), As(III), Cr(III), Ce(IV), Ti(IV), Ge(IV), Fe(III), W(VI), Mo(VI), Se(IV), Pb(II), Ag(I), V(V), Cu(II), Zn(II), Sb(III), Bi(III), In(III), SO_4_^2−^, Cl^−^, CN^−^, HPO_4_^−^, CO_3_^2−^, SCN^−^, CH_3_COO^−^, H_2_PO_4_^−^, CrO_4_^2−^, Br^−^, I^−^, NO_2_^−^, CLO_4_^−^, NH_4_^+^, H_3_PO_4_^−^, and BrO_3_^−^. The possibility of interfering with tin signals in electrochemical methods has been investigated by adding foreign ions to a solution containing a known concentration of tin under optimized conditions. The influence on the occurrence of interference may depend on the working electrode, the base electrolyte, and the complexing agent chosen, but also on the parameters under which the measurements are carried out.

When using the adsorptive stripping voltammetry method with 2,3-dihydroxybenzoic acid (DHBA) as a complexing agent for the determination of tin, interference occurred when germanium ions were added to the test solution. A Sn(IV)–DHBA complex and a Ge(IV)–DHBA complex were accumulated on the working electrode, resulting in a Ge reduction peak on the voltammogram. The peak for germanium appeared at a potential of −0.70 V and for tin, at a potential of −0.52 V, indicating that a 50-fold excess of Ge(IV) had no effect on the tin peak [37].

The use of a solid bismuth microelectrode allowed the determination of tin in the presence of the following foreign ions: a 100-fold excess of Au(III), Cd(II), Co(II), Cr(III), Cr(VI), Cu(II), Ge(IV), Mn(II), Ni(II), Pt(IV), Sb(III), Se(IV), Ti(IV), Tl(I), W(VI), and Zn(II), a 50-fold excess of Fe(III) and Pb(II), as well as a 10-fold excess of Ga(III) and Hg(II). The results obtained did not indicate any reduction in the signal obtained from tin, demonstrating the high selectivity of the developed procedure [42].

In [40], it was found that the signal from tin can be reduced by up to 50% by adding a 5-fold excess of copper to the solution. This is due to the effect of copper on catechols, which causes a reduction in the amount of complexing agent to form a catechol with tin. The recorded voltammograms of a solution containing tin and lead show the presence of a lead peak, but the lead peak is separate from the tin peak. Even high concentrations of lead did not affect the tin signal, allowing the two to be determined simultaneously. Even a 5-fold excess of chromium caused the two tin peaks to overlap, shifting the potential of the second peak towards less negative potentials. The presence of cadmium ions caused the tin peak appearing at a potential of −0.66 V to increase by up to 20%. To remove the interference of copper and cadmium, EDTA should be added to the test solution, which does not affect the tin signals [40].

When tin was determined using the HMDE as the working electrode, the highest signal drop was recorded in the presence of aluminum and copper ions. The lowest signal drop was recorded in the presence of Ni(II) ions at 46%, while the highest was recorded in the presence of Al(III) at 74%. The appearance of the second peak was caused by the addition of cadmium to the solution. The appearance of the cadmium peak on the voltammetric curve is probably related to the reduction of the Cd(II)–catechol coupler formed. The additional peak is well separated from the second stannous peak by about −0.13 V, which does not affect the stannous determination [36].

An optimized method for the determination of Sn(II) and Sn(IV) by differential pulse polarography was tested for the interfering effect of Cu(II), Pb(II), Cd(II), Zn(II), and Fe(III) ions. In this procedure, peaks for Sn(II) appeared at potentials of −0.74 V and −1.17 V. The peaks obtained for cadmium and lead on the voltammogram could overlap with the Sn(II) peak at a potential of −0.74 V. Therefore, for the determination of stannous, the peak at a potential of −1.17 V was chosen so that it would not be affected by any interfering ion. For the determination of Sn(IV), the ions that can interfere with the signal are copper and iron. To eliminate their effect, NaBH_4_ had to be added to the solution [51]. In a later paper [49], EDTA was used to remove the interference that caused the overlap of the tin peak. This was caused by the addition of nickel and indium ions to the solution. The addition of EDTA causes the disappearance of the nickel and lead peaks due to the formation of complexes that are not electroactive under tin determination conditions, but the addition of EDTA does not affect the tin determination. Signal attenuation also occurs in the presence of aluminum ions, which form a complex with tropolone. This interference is prevented by the addition of EDTA [50].

In the case of the potentiometric method, the proposed membrane electrode, which consists of a polymer membrane based on 6-(4-nitrophenyl)-2,4-diphenyl-3,5-diaza-bicyclo[3.1.0]hex-2-ene (NDDBH) as an ionophore, shows very good selectivity for Sn(II) ions compared to other cations and could be used in acidic media. The selectivity coefficient of this electrode increases in the presence of Pb(II), Al(III), and Bi(III) ions. Potential measurements were made of Sn(II) ion concentrations in three additions of Al(III) ion concentrations. The addition of clay was found to cause interference, and these ions were not tolerated in the range of Sn(II) test concentrations. To confirm the effectiveness of the tests carried out, they were repeated for an ion with a low selectivity coefficient. The ion selected was Li(I), and the results obtained showed that the presence of lithium did not excessively affect the membrane potentials [56]. In the work [58], a tin ion-selective electrode consisting of dibenzo-18-crown-6 (DB18C6) as an ionophore in a PVC matrix was developed. When the selectivity of the electrode was tested only for Fe(II) and Hg(II) ions, the selectivity coefficients obtained were high since they were of the order of 10^−2^ or lower for the other cations tested. Iron and mercury ions caused significant interference to the electrode, even when present in quantities comparable to those of tin ions. It was concluded that an increase in the concentration of Fe(II) ions would result in a decrease in the detection limit and linear range of tin determination [58]. A tin(II) ion-selective electrode consisting of dibenzo-18-crown-6 (DB18C6) as an ionophore in a PVC matrix was developed. Under laboratory conditions, the detection limit of the tin assay was 8.0 × 10^−7^ mol L^−1^. In order to verify the correct functioning of the electrode, the determination of tin in synthetic samples was carried out. Known amounts of Sn(II) were added to solutions containing Pb(II), Al(III), and Fe(II). The results obtained confirm the possibility of determining tin in the presence of lead and aluminum ions, but in the presence of iron, the results obtained are overestimated. The proposed electrode allows for the determination of tin with high sensitivity and good selectivity, except in the presence of Fe(II) [59].

In electrochemical methods used to determine tin in environmental samples, interference may occur due to the presence of organic compounds. Surfactants and humic compounds are organic substances that can accumulate on the surface of the working electrode and thus block the space for the analyte being determined. The surfactant that was most frequently chosen to test the procedure for interference was Triton X–100 [35,36,37,40,41,42,43]. In the works [35,40], a decrease in the tin signal from 40% to 90% was reported. A smaller effect of Triton X–100 on the tin signal was noted when using a fixed bismuth microelectrode [42] and a hanging drop mercury electrode [37] as the working electrode. The suppression of peak tin currents indicates that the organic substance was more strongly adsorbed on the surface of the working electrode. To eliminate this interference effect, the sample should be irradiated with UV rays before analysis. Heppeler et al. [41] proposed another method to remove the signal attenuation by Triton X–100. They concluded that changing the accumulation potential of the tin–chloranilic acid complex in the presence of 2.5 mg L^−1^ would allow for the determination of tin, although the sensitivity would be lower. Adamczyk et al. analyzed other substances that could interfere with the signal. The interference effect was tested in the presence of an anionic surfactant (SDS), a cationic surfactant (CTAB), a biosurfactant (Rhamnolipid), and humic substances such as humic acids (HAs) and fulvic acids (FAs). The results obtained show that these substances do not interfere with the determination of tin when their solution concentration is up to 5 mg L^−1^ since the tin signal remains practically unchanged. A reduction in the tin peak of up to 20% was observed when 7 mg L^−1^ CTAB and 6 mg L^−1^ Triton were added to the solution. In contrast, the smallest reduction in tin signal was observed after the addition of up to 15 mg L^−1^ Rhamnolipid [42].

## 5. Simultaneous Determination of Tin with Other Ions

Electrochemical method analysis is a common and accurate analytical technique for determining the content of different elements in samples. However, the simultaneous determination of several elements by a single electrochemical method can be difficult due to overlapping signals from other metals and inter-element interference. In summary, developing methods for the simultaneous determination of two or more elemental ions requires the appropriate choice of technique, electrodes, and measurement conditions, as well as consideration of potential inter-element interference. In the case of tin, there are mainly works devoted to the simultaneous determination of tin and lead [21,23,40,47], whereas in one case, in addition to tin and lead, molybdenum was also determined [53]. One procedure for the simultaneous determination of tin and zinc has also been described in the literature [38].

Dirilgen et al. demonstrated the feasibility of the simultaneous determination of tin and lead ions using anodic stripping voltammetry. The resulting peaks on the voltammogram were well separated as the tin peak was recorded at a potential of –0.60 V and the lead peak at a potential of −0.45 V. In order to adequately separate the tin and lead peaks, a base electrolyte was chosen to ensure that the peaks were well-shaped and far apart from each other. Experiments were carried out using two base electrolytes, one consisting of cetyltrimethylammonium bromide (CTAB) in a mixture of citrate and oxalate buffer and the other consisting of methylene blue in oxalate buffer. The use of CTAB, which was part of the first base electrolyte, did not give the desired results; the peak recorded from the tin was asymmetric. In contrast, when methylene blue and oxalate buffer were present in the solution, the recorded voltammograms gave well-defined tin and lead peaks that were symmetrical and reproducible. The optimized method was used for the simultaneous determination of tin and lead in samples found by archaeologists. This research was intended to demonstrate the technological capabilities of early metallurgists [23]. In another procedure, the simultaneous determination of lead and tin ions was carried out using a glassy carbon mercury film electrode (GCMFE) as the working electrode. The measurements were carried out by differential pulse cathodic voltammetry with formation of a tin–catechol complex and direct reduction of lead(II) ions. This method was successfully applied to the determination of tin and lead in the presence of a 4000-fold excess of lead ions. It has also been shown that in the absence of lead ions, saturation of the GCMFE electrode surface with the tin–catechol complex occurs more slowly than in the presence of lead ions [40]. In the work [48], a simple and sensitive method using differential pulsed polarography was developed for the simultaneous determination of tin and lead. Due to the overlapping of the polarographic waves of tin and lead, it was decided to optimize the polarographic method for the simultaneous determination of lead and tin. The ability to determine both lead and zinc ions in a single measurement cycle was achieved in the concentration range of 0.05 mg L^−1^ to 3.50 mg L^−1^. To demonstrate the effectiveness of the developed method, tin and lead were determined in a zinc battery cell. The standard addition recovery was 97.9% for tin and 100.5% for lead, demonstrating the suitability of the developed procedure for these ions [48].

Differential pulse polarography was used for the simultaneous determination of tin, lead, and molybdenum. In an appropriately selected basic electrolyte, two peaks of Sn(II), one peak of Sn(IV), two peaks of Mo(VI), and one peak of Pb(II) appeared on the polarogram. The appearance of six peaks on the polarogram is when the base electrolyte consists of 0.05 mol L^−1^ EDTA and 0.2 mol L^−1^ NaOAc, while the pH of the solution is 3.5. However, if the pH of the solution is increased to 5.5, the signal from lead disappears and a peak from molybdenum is missing. This shows the importance of the choice of the base electrolyte for the signal obtained [53].

HBO was used as a complexing agent for the simultaneous determination of Sn(II) and Zn(II) on a hanging drop mercury electrode. The signal was obtained by reducing the complexes adsorbed on the surface of the working electrode. Two well-separated peaks appeared on the voltammogram, the tin peak appearing at a potential of about −0.35 V and the zinc peak at a potential of about –1.0 V. The developed method was used for the determination of tin and zinc in biological samples, and satisfactory results were obtained, confirming the usefulness of the developed procedure [38].

## 6. Application

The developed analytical procedures using electrochemical methods have found application for the determination of tin in a wide range of real samples. Table 4 and Table 5, which summarize the practical application of the discussed methods in environmental and real samples, allow us to assess the suitability of the developed methods for the determination of tin. Tin ions have been tested in food samples, synthetic samples with known levels of tin, different types of alloys, water samples taken from different locations, and wastewater samples.

**Figure 4:**
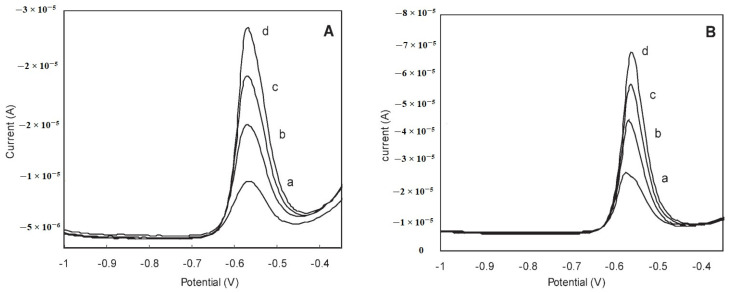
Measurement of tin in unspiked (**A**) and spiked (**B**) samples of pineapple juice. Voltammograms show the sample (a) and three standard additions (b–d) of 10 µg L^−1^ tin (**A**) and 20 µg L^−1^ tin. (**B**) Supporting electrolyte: 2.5 mol L^−1^ NaBr, deposition potential −1.3 V, deposition time: 240 s. Square-wave voltammetric stripping scan with a frequency of 40 Hz, amplitude of 30 mV, and a step height of 7 mV. A 30 s cleaning step at +0.3 V while stirring was applied to the electrode between measurements. After re-elaboration from Ref. [25] and permission from the publisher.

The optimized procedure for the determination of tin by anodic stripping voltammetry using a bismuth membrane electrode described in [25] was used for the determination of tin in pineapple juice. Prior to the measurements, the pineapple juice sample was diluted 100-fold and prepared in a solution of 2 mol L^−1^ HCl. Measurements were carried out on enriched and unenriched samples to which known amounts of Sn(IV) were added. The voltammograms obtained show the Sn(IV) reduction peaks (Figure 4). The Sn content determined was 4.98 mg L^−1^, with a recovery of 99.6% [25].

It is possible to determine the concentration of tin in food and wastewater samples using the ASV method. Yi-Heng Li et al. decided to test the developed method for the determination of tin in real samples. They chose canned foods such as fermented bean curd, lychee juice, orange juice, and milk tea as well as wastewater. All samples were properly prepared before measurements were taken. The results obtained are shown in Table 4. In order to compare the results obtained with the ASV method, a reference method was employed, in this case, graphite furnace atomic absorption spectrometry (GF–AAS). The data obtained confirm the suitability of the developed method for practical use [37].

A highly selective and sensitive method for the simultaneous determination of traces of tin and zinc was used to determine the concentrations of these elements in biological samples. Human hair and blood serum were chosen as biological samples. The method is based on the adsorptive accumulation of Sn(II) and Zn(II) complexes formed with 2,2-hydroxyphenylbenzoxazole (HBO) on the surface of the HMDE electrode. A template addition method was used to eliminate the matrix effect. The results obtained are shown in Table 5 where it can be seen that the recovery of Sn(II) was in the range of 91.1% to 104.06%, whereas that of Zn(II) was in the range of 99.3% to 104.5% [38].

Adsorptive stripping voltammetry was proposed for the determination of tin concentrations in environmental samples. Sea and river water samples were determined by the standard addition method. The results obtained were reliable, which led to the conclusion that the developed method is useful for the determination of tin in environmental samples. The standard addition method was also used for the determination of tin in lake sediment. The tin content in the sediment calculated by the AdSV method was found to be 66.00 ± 1.2 ng g^−1^, but to confirm this result, the determination of tin in the sediment was carried out by hydride generation atomic absorption spectrometry [36].

Wang et al. proposed a method for the determination of traces of tin using tropolone as a complexing agent. The optimized method was used for the determination of tin in river water and orange juice samples. In order to determine the actual concentration of tin in these samples, calibration curves were constructed with increasing concentrations of tin. The resulting graphs were linear, and the correlation coefficient was 0.999. The same tests were carried out on a cola drink taken from a can. The recorded voltammograms showed a well-shaped peak from tin, indicating the presence of tin in this drink [34].

In [42], the developed method for the determination of tin was validated, and real samples were analyzed for the presence of tin. Two certified reference materials, SPS–WW1 wastewater and SPS–SW1 surface water, were used for validation. The reference materials did not contain Sn(II), whereas SPS–WW1 contained 13 elements with known concentrations and SPS–SW1 contained 43 elements, as confirmed by the certificates. The measurements were carried out using the standard addition method, and the results obtained are given in Table 5. However, in order to demonstrate the applicability of the proposed method, tests were carried out on water samples from the Bystrzyca River and rainwater. The recorded voltammeters for these samples showed no signal for tin, so the samples were fortified with Sn(II). In addition, to demonstrate the suitability and accuracy of the proposed method for real water samples, water from the Bystrzyca River and rainwater were selected for analysis. However, the voltammograms obtained for both samples did not show any tin signal, indicating that the samples analyzed were enriched in Sn(II). A relative standard deviation ranging from 3.9% to 6.2% was obtained, which shows the satisfactory accuracy of the proposed method and clearly demonstrates the suitability of this procedure for the determination of Sn(II) in various environmental samples [42].

The developed ion-selective electrodes for the determination of tin have been tested on different types of real samples. In the paper [60], a Sn(II) ion-selective electrode prepared with a polymeric membrane based on 6-(4-nitrophenyl)-2,4-diphenyl-3,5-diazabicyclo[3.1.0]hex-2-ene (NDDBH) as an ionophore was used. It was applied as an indicator electrode for the potentiometric determination of Sn(II) ions in real samples. These samples were synthetic solutions containing tin, a copper-based alloy, a Sn–Pb alloy, canned meat, and industrial waste. They were also subjected to tin determination by AAS. The results obtained are shown in Table 4. By comparing the results obtained with the two methods, it can be seen that the electrode can be successfully used in biological, industrial, and environmental applications for Sn(II) monitoring [60]. Hosseini et al. proposed the potentiometric determination of tin(II) tris(3-(2-hydroxybenzophenone)propyl)amine (THPA) as an ionophore in a PVC matrix. This sensor has been used for the determination of tin in synthetic solutions, industrial effluents, canned fish, orange juice, and grape juice. Table 4 shows the results obtained using the potentiometric method and the reference method, in this case, ICP [58].

A paper has appeared in the literature on the determination of tin(II) using coulometric stripping potentiometry (SPC). This optimized procedure was used to determine tin(II) in a jewelry alloy based on gold, silver, platinum, and copper. According to the certificate, the alloy contained approximately 1% tin. Using the proposed procedure, the authors determined the tin content to be 1.56 ± 0.02% with a relative standard deviation of 7% [61].

## 7. Conclusions

In conclusion, this overview has focused on the use of electrochemical methods for the determination of tin. The literature contains works, in which the techniques of stripping voltammetry, potentiometry, and polarography, are used for the determination of tin. The review paid attention to the selection of the basic electrolyte. The working electrodes used in the determination of tin are presented together with the detection limits obtained. Interference effects caused by the presence of foreign ions and organic substances are discussed. Ions that interfered with the tin signal include germanium, lead, copper, aluminum, and cadmium. Their influence on the signal was most often related to the appearance of an additional peak in the voltammogram. In the case of ion-selective electrodes used in the determination of tin, the interfering ions affecting the selectivity of the electrode were iron(II), lead(II), bismuth(III), and aluminum(III). Few studies have examined the influence of surfactants on the tin signal; most often, these studies were conducted in the presence of Triton X–100. Only one study examined a group of humic substances, which did not negatively affect the tin signal. Works on the simultaneous determination of tin with other elements are also discussed. Lead was the most common foreign ion selected for simultaneous determination because its peak appeared close to that of tin. Procedures have also been developed to determine tin together with elements such as zinc and molybdenum. The last chapter of the article contains information on the use of the developed procedures for the determination of tin in real samples. Their practical application has been demonstrated in studies conducted on food samples, environmental samples, synthetic solutions, and biological samples. The obtained results for tin determination confirm the possibility of using these methods to analyze real samples.

## Figures and Tables

**Table 1:** Analytical performance of procedures for the determination of tin by the voltammetry method.

Method	Working Electrode	Supporting Electrolyte	Complexing Agent	Linear Range [mol L^−1^]	Detection Limit [mol L^−1^]	Accumulation Time [s]	Investigated Interferents	Application	Ref.
AdSV	HMDE	(pH 2.1)	tropolone	no data–4.0 × 10^−9^	5.0 × 10^−12^	600	In(III), Ni(II), Cr(VI), Al(III), As(III), V(V), Zn(II), Se(IV), Zr(II), W(VI), Bi(III), U(VI), Ba(II), Te(IV), Ti(IV), Mn(II), Fe(III), Mo(VI) Triton X–100	sea water, estuarine water	[35]
AdSV	HMDE	0.1 mol L^−1^ acetate buffer (pH 4.2)	catechol	0–1.3 × 10^−8^	4.2 × 10^−11^	180	Ni(II), Co(II), Fe(III), Al(III), Cu(II), Cd(II) EDTA, Triton X–100	water, sediment samples	[36]
AdSV	HMDE	0.1 mol L^−1^ formate buffer (pH 3.1)	DHBA	8.4 × 10^−11^–3.4 × 10^−7^	4.2 × 10^−11^	60	Ca(II), Mg(II), Al(III), Zn(II), Mn(II), Co(II), As(III), Be(II), Fe(III), Ni(II), Ga(III), Cu(II), Pb(II), Bi(III), Cd(II), Hg(II), Sb(III), Ti(III), In(III), W(VI), Cr(VI), Se(IV), Mo(VI), V(V), Ge(IV) Triton X–100	canned food (fermented bean curd, grape juice, milk tea), human hair, waste water	[37]
AdSV	MFE	0.01 mol L^−1^ acetate buffer (pH 4.3)	chloranilic acid	4.2 × 10^−9^–1.7 × 10^−7^	1.9 × 10^−11^	400	Sb(III), V(V), Fe(III), W(VI) Triton X–100, HA	drainage water	[41]
CSV	CME	0.01 mol L^−1^ sodium chloride (pH 1.9)	–	8.4 × 10^−10^–7.6 × 10^−9^	8.4 × 10^−10^	180	K(I), Ca(II), Ba(II), Tl(I), Al(III), Bi (III), Cr(III), Ce(IV), Co(II), Fe(III), As(V), V(V), Ag(I), Mg(II), Cd(II), Hg(II), Pb(II), Ge(IV), Zn(II), Cu(II), Se(IV), Ni(II), Te(III), NO_3_^−^, CH_3_COO^−^, SCN^−^, H_2_PO_4_^−^, SO_4_^2−^, Cr_2_O_7_^2−^	human hair	[33]
AdSV	HMDE	0.1 mol L^−1^ acetate buffer (pH 5.0)	HBO	1.7 × 10^−9^–8.4 × 10^−7^	7.6 × 10^−10^	90	Na(I), Cs(I), Ag(I), Al(III), Fe(III), Co(II), Mn(II), Cr(III), Cd(II),Pb(II) Sn(IV), Li(I), Ni(II), Cu(II), Mg(II), NH_4_^+^, Br^−^, Cl^−^, SCN^−^, H_3_PO_4_^−^, NO_2_^−^, CLO_4_^−^, SO_3_^2−^, BrO_3_^−^, I^−^	blood serum, human hair	[38]
ASV	CPE	0.1 mol L^−1^ acetate buffer (pH 4.5)	BPR	8.4 × 10^−10^–4.2 × 10^−7^	5.0 × 10^−10^	120	Ca(II), Mg(II), Al(III), Zn(II), Ba(II), Co(II), Hg(II), Mn(II), Cd(II), Ga(III), As(III), Cr(III), Ti(IV), Ge(IV), Fe(III), W(VI), Mo(VI), Se(IV), Pb(II), Ag(I), V(V), Sb(III), Cu(II)	canned food (fermented bean curd lychee juice orange juice milk tea), waste water	[24]
AdSV	HMDE	0.1 mol L^−1^ acetate buffer (pH 4.3)	oxine	–	3.5 × 10^−10^ 7.0 × 10^−11^	60 300	Zn(II), Mn(II), Co(II), Fe(II), Fe(III), Ni(I), Al(III), Ga(III), Hg(II); Cr(III), Cu(II), V(V), Ce(IV), Bi(III), Ag(I), Pb(II), Cd(II), In(III), W(VI), Mo(VI), Cd(II)	blood, serum, plants (orange, grass, corn)	[39]
AdSV	CNTs/SGC	0.1 mol L^−1^ acetate buffer (pH 5.7)	cupferron	1.0 × 10^−9^–1.0 × 10^−7^	3.1 × 10^−10^	95	Au(III), Cd(II), Pt(IV), Se(IV), Zn(II), Fe(III), Pb(II), Hg(II), V(V), In(III), Ti(IV), Mo(VI), Pt(IV) SDS, CTAB, Triton X–100, Rhamnolipid, HA, FA	CRM (SPS–WW1 Waste Water), river water, tap water	[43]
AdSV	HMDE	0.1 mol L^−1^ acetate buffer (pH 4.0)	tropolone	–	2.3 × 10^−10^	560	–	river water, orange juice	[34]
ASV	modified thick film electrodes	1.0 mol L^−1^ hydrochloric acid	–	8.4 × 10^−9^–8.4 × 10^−7^	7.6 × 10^−9^	60	Cd(II), Pb(II), Cu(II), Zn(II), Tl(III) Triton X–100, HA	canned fruit juices (orange, pineapple, orange with pulp)	[32]
ASV	Bi/poly(BPB)GCE	1.0 mol L^−1^ hydrochloric acid	–	2.0 × 10^−8^–2.0 × 10^−5^	7.0 × 10^−9^	60	Na(I), K(I), Ca(II), Mg(II), Mn(II), Ni(II), Co(II), Hg(II), Zn(II), Pb(II), Cu(II), Cd(II), Fe(III), SO_4_^2−^, Cl^−^	canned fruit juice (pineapple, orange, grape, mango),	[30]
AdSV	GCMFE	0.1 mol L^−1^ acetate buffer (pH 4.2–4.7)	catechol	0–2.9 × 10^−8^	4.2 × 10^−9^	300	Ca(II), Zn(II), Fe(III), Ni(II), Mg(II), Pb(II), Cu(II), Cd(II)	canned fruit juice (pineapple juice, apricot nectar, grapefruit juice, tomato juice, peach nectar)	[40]
ASV	BiFE	0.1 mol L^−1^ acetate buffer (pH 4.5)	–	8.4 × 10^−9^–8.4 × 10^−7^	2.2 × 10^−9^	60	–	sea water	[26]
AdSV	BiFµE	0.1 mol L^−1^ acetate buffer (pH 4.6)	cupferron	8.0 × 10^−9^–8.0 × 10^−7^	2.1 × 10^−9^	40	Au(III), Cd(II), Co(II), Cr(III), Cr(VI), Cu(II), Ge(IV), Mn(II), Ni(II), Pt(IV), Sb(III), Se(IV), Ti(IV), Tl(I), W(VI), Zn(II), Fe(III), Pb(II), Ga(III), Hg(II) SDS, CTAB, Triton X–100, Rhamnolipid, HA, FA	CRM (SPS–WW1 Waste Water, SPS –SW1 Surface Water), river water, rain water	[42]
ASV	CNT	borate buffer (pH 7.5)	–	2.5 × 10^−9^–2.1 × 10^−6^	1.0 × 10^−9^	240	K(I), Li(I), Ag(I), Mg(II), Ca(II), Ba(II), La(III), Cr(III), Fe(II), Fe(III), Na(I), Al(III), Co(II), Ti(III), Cd(II), Cu(II), Zn(II), HPO_4_^−^, CO_3_^2−^, CN^−^	fruit juice, bottled water	[31]
ASV	BiFE	0.1 mol L^−1^ acetate buffer (pH 4.5)	caffeic acid	5.0 × 10^−8^–5.0 × 10^−6^	1.8 × 10^−8^	90	–	–	[29]
ASV	BiFE	75 mmol L^−1^ oxalic acid, 75 µmol L^−1^ CTAB	–	4.2 × 10^−8^–2.1 × 10^−6^	1.6 × 10^−8^	120	Cu(II), Se(IV), Mn(II), Fe(III), Mo(VI), V(V), Cr(III), Ni(II), Sb(III), Zn(II), Bi(III)	canned fruit juice (pineapple)	[27]
ASV	BiFE	0.1 mol L^−1^ acetate buffer (pH 4.5)	caffeic acid	1.7 × 10^−7^–7.83 × 10^−6^	1.4 × 10^−7^	90	–	biodiesel	[28]
ASV	BiFE	2.5 mol L^−1^ sodium bromide	–	2.0 × 10^−5^–2.0 × 10^−4^	–	120	–	canned fruit juice	[25]

HMDE—hanging drop mercury electrode, MFE—mercury film electrode, CME—chemically modified electrode, CNTs/SGC—multiwall carbon nanotubes/spherical glassy carbon, CNT—multiwall carbon nanotubes, BiFE—bismuth film electrode, CPE—carbon paste electrode, Bi/poly(BPB)GCE—bismuth/poly 92 (bromophenol blue) modified glassy carbon electrode, GCMFE—glassy carbon mercury film electrode, BiFµE—solid bismuth microelectrode, DHBA—3,4-dihydroxybenzoic acid, HBO—2,2-hydroxyphenylbenzoxazole, BPR—bromopyrogallol red, oxine—8-hydroxyquinoline.

**Table 2:** Polarographic techniques for tin determination.

Method	Working Electrode	Conditions (Supporting Electrolyte, pH, Temperature)	Linear Range [mol L^−1^]	Detection Limit [mol L^−1^]	Investigated Interferents	Application	Ref.
DPP	DME	0.2 mol L^−1^ sodium acetate/acetic acid buffer of pH 4.7, 8.2 × 10^−4^ mol L^−1^ tropolone	8.4 × 10^−9^–4.2 × 10^−5^	8.4 × 10^−10^	Al(III), As(V), Be(II), Bi(III), Ca(II), Cd(II), Co(II), Cr(II), Cu(II), Fe(III), Ga(III), In(III), V(V), W(VI), Ti(IV), Mo(VI)	river rhine, fruit juice (pineaplle)	[50]
SSP	DME	0.5 mol L^−1^ oxalic acid, 0.02% methylene blue	Sn(IV) 1.7 × 10^−7^–8.4 × 10^−6^	8.4 × 10^−8^	Cd(II), Pb(II), Fe(II), Ag(I), Zn(II), Ca(II), Mg(II), HCO_3_^−^, Br^−^, CO_3_^2−^	canned fruits (pineapple, orange, pear)	[51]
DPP	HMDE	methanol, perchloric acid	Sn(II) 8.4 × 10^−6^–4.2 × 10^−4^	4.2 × 10^−8^	–	–	[54]
DPP	DME	0.1 mol L^−1^ NaOH, 0.1 mol L^−1^ KNO_3_	–	Sn(II) 5.5 × 10^−7^ Sn(IV) 8.2 × 10^−7^	Fe(III), Cu(II), Pb(II), Cd(II), Zn(II),	canned tomato sauce	[52]
DPP	DME	0.05 mol L^−1^ EDTA, 0.2 mol L^−1^ NaOAc, pH 3.5	Sn(IV) 2.0 × 10^−6^–1.0 × 10^−5^	Sn(IV) 3.0 × 10^−7^	–	shipyard water, canned tomato sauce	[53]
DPP	SMDE	0.1 mol L^−1^ catechol, 0.1 mol L^−1^ NaCIO_4_, 0.1 mol L^−1^ HCIO_4_	Sn(IV) 8.4 × 10^−6^–8.4 × 10^−5^	2.4 × 10^−7^	–	–	[47]
DPV	HMDE	0.25 mol L^−1^ HCl, 5.1 × 10^−7^ mol L^−1^ Pt	4.2 × 10^−6^–8.4 × 10^−6^	2.5 × 10^−6^	Ir(III), Pd(II), Rh(II), Ru(I), Re(I), Pt(IV)	–	[57]
DPP	gold amalgamated electrode	3.5 mol L^−1^ HCl	4.2 × 10^−6^–1.9 × 10^−3^	1.3 × 10^−6^	Zn(II), Mo(VI), Mn(II), Cr(VI), Se(VI), Ti(VI), Ga(II), Al(III), U(VI), V(V), Te(IV), Bi(III), Fe(III) Rh(III), Ru(III), Cd(II), Pd(II), Os(VIII), Co(II), Ni(II), Cu(II), Pb(II), Cr(VI), Sb(III), Tl(I), CH_3_COO^−^, NO_3_^−^, NH_4_^+^, F^−^, Br^−^, SCN^−^, I^−^, Cl^−^, CO_3_^2−^, EDTA	fly ash, soil, sediment river, industrial effluents	[56]
DPP	SMDE	0.1 mol L^−1^ NaOH, 0.5 mol L^−1^ sodium malonate, pH 9.8	Sn(IV) 8.4 × 10^−5^–3.4 × 10^−4^	1.3 × 10^−5^	–	–	[49]
DPP	SMDE	0.1 mol L^−1^ HCl, 0.01 mol L^−1^ KSCN	Sn(II) 4.2 × 10^−7^–2.9 × 10^−5^	–	–	zinc chamber of battery	[48]
DPP	HMDE	0.2 mol L^−1^ HCl, 0.2 mol L^−1^ citric acid	0–1.0 × 10^−3^	–	–	–	[55]

DPP—differential pulse polarography, SSP—single–sweep polarography, DPV—differential pulse voltammetric, DME—dropping mercury electrode, HMDE—hanging mercury drop electrode, SMDE—static mercury drop electrode.

**Table 3:** Characteristics of ion-selective electrodes for Sn(II) determination.

Membrane Composition	pH	Concentration Range [mol L^−1^]	Detection Limit [mol L^−1^]	Response Time [s]	Life Time [Days]	Interference	Application	Ref.
PVC–NDDBH	1.0	1.0 × 10^−5^–1.0 × 10^−1^	4.0 × 10^−6^	20	42	Pb(II), Bi(III), Al(III), Ca(II), Co(II), Zn(II), Cd(II), Ba(II), Mg(II), Ni(II), Cu(II), Cr(III), Li(I), Na(I), Tl(I), Cs(I), NH_4_^+^	synthetic solutions, standard copper–based alloy, Sn–Pb alloy, industrial wastewater, Canned meat	[60]
PVC–THPA	3.0	5.0 × 10^−7^–1.0 × 10^−1^	2.0 × 10^−7^	˂15	56	Ag(I), Tl(I), Pb(II), Mg(II), Na(I), Cu(II), K(I), Cs(I), Bi(III), Hg(II), Co(II), Cd(II), Al.(III), Ca(II), Cr(III), La(III), Ce(III), Ni(II)	synthetic solutions, industrial wastewater, orange juice, grapes juice, canned fish	[58]
PVC–DB18C6	1.0	1.0 × 10^−6^–1.0 × 10^−2^	8.0 × 10^−7^	˂15	90	Al(III), Mn(II), Mg(II), Zn(II), Cu(II), Co(II), Fe(II), Cd(II), Ca(II), Pb(II), Hg(II), Sr(II), Bi(III), Fe(III)	alloy Sn–Pb	[59]

PVC—poly(vinyl chloride), DB18C6—dibenzo-18-crown-6, NDDBH—6-(4-nitrophenyl)-2,4-diphenyl-3,5-diaza-bicyclo [3.1.0]hex-2-ene, THPA—tris(3-(2-hydroxybenzophenone)propyl)amine.

**Table 4:** Tin recovery values obtained by stripping voltammetry on real samples.

Method	Sample	Added Sn(II) [mg L^−1^]	Added Pb(II) [mg L^−1^]	Found Sn(II) [mg L^−1^]	Found Pb(II) [mg L^−1^]	Ref.
ASV	Orange juice	–	–	0.372	0.118	[32]
		0.5	0.1	0.875	0.231	
	Pineapple juice	–	–	0.283	0.099	
		0.25	0.1	0.535	0.189	
	Orange with pulpe drink	–	–	237.0	0.25	
		200.0	0.2	444.0	0.53	
Method	Sample	Add Sn(II) [mol L^−1^]		Found Sn(II) [mol L^−1^]		Ref.
ASV	Pineapple juice	3 × 10^−7^		4.19 × 10^−7^		[30]
		1 × 10^−6^		11.03 × 10^−7^		
		3 × 10^−6^		32.41 × 10^−7^		
		8 × 10^−6^		78.84 × 10^−7^		
	Orange juice	3 × 10^−7^		7.43 × 10^−7^		
		1 × 10^−6^		14.82 × 10^−7^		
		3 × 10^−6^		33.53 × 10^−7^		
		8 × 10^−6^		85.87 × 10^−7^		
	Grape juice	3 × 10^−7^		5.93 × 10^−7^		
		1 × 10^−6^		12.69 × 10^−7^		
		3 × 10^−6^		31.85 × 10^−7^		
		8 × 10^−6^		84.01 × 10^−7^		
	Mango Juice	3 × 10^−7^		8.54 × 10^−7^		
		1 × 10^−6^		17.79 × 10^−7^		
		3 × 10^−6^		37.02 × 10^−7^		
		8 × 10^−6^		86.13 × 10^−7^		
Method	Sample	Added Sn(II) [µg L^−1^]	Added Zn(II) [µg L^−1^]	Found Sn(II) [µg L^−1^]	Found Zn(II) [µg L^−1^]	Ref.
AdSV	Blood Setum	0	0	10.81	17.0	[38]
		10	10	19.92	27.2	
		15	15	26.42	31.9	
	Human Hair	0	0	14.65	22.55	
		10	10	24.0	33.0	
		15	15	30.0	37.9	
Method	Sample	Added Sn(II) [ng mL^−1^]		Found Sn(II) [ng mL^−1^]		Ref.
ASV	Fruit juice	–		0		[31]
		99.5		10		
		93.45		20		
	Bottled water	0.63		–		
		98.2		10		
		100.4		20		
Method	Sample	Added Sn [mg L^−1^]		Found Sn [mg L^−1^]		Ref.
AdSV	Pineapple juice	10.00		10.83		[40]
	Apricot nectar	5.00		3.18		
	Grapefruit juice	10.00		9.46		
	Tomato juice	5.00		3.23		
	Peach nectar	10.00		10.19		
Method	Sample	Added Sn(IV) [ng g^−1^]		Found Sn(IV) [ng g^−1^]		Ref.
CSV	Human hair					[33]
	I	50		50.85		
	II	50		49.10		
	II	50		48.55		
Method	Sample	Sn(II) added [nmol L^−1^]		Found Sn(II) [nmol L^−1^]		Ref.
AdSV	SPS–WW1 Waste Water	30.0		29.0		[42]
		80.0		83.0		
		160.0		169.0		
	SPS–SW1 Surface Water	30.0		28.7		
		80.0		84.6		
		160.0		153.0		
	Bystrzyca river water	30.0		31.0		
		80.0		76.0		
		160.0		167.5		
	Rain water	30.0		30.8		
		80.0		87.0		
		160.0		151.5		
Method	Sample	Sn(II) Added [nmol L^−1^]		Found Sn(II) [nmol L^−1^]		Ref.
AdSV	SPS–WW1 Waste Water	–		–		[43]
		5.00		5.35		
		10.00		10.34		
	Bystrzyca river water	5.00		4.77		
		10.00		9.55		
	Tap water	5.00		5.15		
		10.00		10.23		

**Table 5:** Application of electrochemical methods for tin determination and comparison with references.

Sample	Found Sn(II) by ASV Method [µg g^−1^]	Found Sn(II) by GF–AAS Method [µg g^−1^]		Ref.
Fermented bean curd	48.3	46.7		[24]
Lychee juice	41.9	43.7		
Orange juice	23.1	22.3		
Milk tea	8.8	9.3		
Sample	Found Sn(II) by ASV Method [×10^−5^ mol L^−1^]	Found Sn(II) by GF–AAS Method [×10^−5^ mol L^−1^]		Ref.
Pineapple juice	1.14 ± 0.05	1.17 ± 0.05		[30]
Orange juice	4.51 ± 0.20	4.47 ± 0.18		
Grape juice	2.82 ± 0.11	2.86 ± 0.10		
Mango Juice	0.750 ± 0.03	7.33 ± 0.03		
Sample	Found Sn(IV) by AdSV Method [mg L^−1^]	Found Sn(IV) by AAS Method [mg L^−1^]		Ref.
Pineapple juice	100.3 ± 10.9	96.9 ± 3.4		[40]
Apricot nectar	7.7 ± 2.5	7.16 ± 1.14		
Grapefruit juice	54.2 ± 5.4	67.56 ± 1.53		
Tomato juice	51.0 ± 5.1	58.17 ± 1.46		
Peach nectar	173.5 ± 14.1	201.8 ± 6.6		
Sample	Found Sn by AdSV Method [µg L^−1^]	Found Sn by AAS Method [µg L^−1^]		Ref.
Drainage water 1	38.5 ± 2.5	44		[41]
Drainage water 2	88 ± 4.8	90		
Sample	Found Sn(IV) by AdSV Method [µg g^−1^]	Found Sn(IV) by GF–AAS Method [µg g^−1^]		Ref.
Fermented bean curd	39.4	37.9		[37]
Grape juice	25.4	26.5		
Milk tea	10.8	11.5		
Human hair	[ng g^−1^]	[ng g^−1^]		
A	58.3	56.9		
B	47.3	49.1		
C	28.7	29.9		
Waste water	[µg L^−1^]	[µg L^−1^]		
A	5.1	5.5		
B	3.3	2.9		
C	1.2	0.9		
Sample	Found Sn(IV) by Single–sweep polarography Method [µg mL^−1^]	Found Sn(IV) by Spectrophotometric Method [µg mL^−1^]		Ref.
Pineapple juice	2.30 ± 0.23	2.40 ± 0.21		[51]
Orange juice	8.50 ± 0.41	8.20 ± 0.52		
Pear juice	0.50 ± 0.05	8.20 ± 0.52		
Sample	Found Sn(IV) by DPP Method [µg g^−1^]	Found Sn(IV) by AAS Method [µg g^−1^]		Ref.
Fly ash near Indraprastha	5.26 ± 0.03	5.25 ± 0.06		[56]
Soil near Wazirpur industrial area	13.45 ± 0.06	13.48 ± 0.08		
Sediment of Yamuna river	2.45 ± 0.04	2.44 ± 0.05		
Industrial effluents from electroplating and ceramic industry	310 ± 4 µg mL^−1^	312 ± 3 µg mL^−1^		
Sample	Found Sn by DPP Method [ng mL^−1^]	Found Sn by AAS Method [ng mL^−1^]	Found Sn by ASV Method [ng mL^−1^]	Ref.
River Rhine	12.3 ± 0.7	13.1 ± 0.8	12.0 ± 0.5	[50]
Pineapple juice	668 ± 13	680 ± 21	695 ± 15	
Sample	Found Sn(II) by Potentiometry Method [µg g^−1^]	Found Sn(II) by ICP Method [µg g^−1^]		Ref.
Synthetic solution	[mg mL^−1^]	[mg mL^−1^]		[58]
(2.2% Co^2+^, 1.5% Cr, 0.6% Sn)	5.63 ± 0.11	6.12 ± 0.04		
(2.0% Fe, 1.5% Cd, 0.5% Sn)	4.83 ± 0.20	5.10 ± 0.03		
(1.0% Al, 2.0% Cu, 0.5% Sn)	4.75 ± 0.15	5.08 ± 0.08		
Wastewater sample	300.7 ± 2.2	299.5 ± 0.4		
Orange juice	50.3 ± 0.1	49.5 ± 0.1		
Canned fish	100.5 ±0.1	99.7 ± 0.1		
Grape juice	58.3 ± 0.3	59.0 ± 0.1		
Sample	Found Sn(II) by Potentiometric Method	Found Sn(II) by AAS Method		Ref.
Synthetic solutions				[60]
(1.2% Ni, 0.5% Sn)	5.37 ± 0.06 mg mL^−1^	5.12 ± 0.03 mg mL^−1^		
(1.5% Fe, 0.5% Cr, 0.4% Sn)	4.23 ± 0.12 mg mL^−1^	4.09 ± 0.08 mg mL^−1^		
Standard copper–based alloy	3.7 ± 0.2 mg g^−1^	3.8 ± 0.1 mg g^−1^		
Canned meat	281.7 ± 0.6 µg g^−1^	282.4 ± 0.2 µg g^−1^		
Sn–Pb alloy	625.4 ± 0.7 mg g^−1^	632.2 ± 0.4 mg g^−1^		
Industrial wastewater	310.5 ± 2.3 mg mL^−1^	311.8 ± 1.2 mg mL^−1^		

## Data Availability

Not applicable.

## References

[1] Habashi F. (2013). Tin, Physical and Chemical Properties. Encyclopedia of Metalloproteins.

[2] Smith P.J. (2012). Chemistry of Tin.

[3] Gielen M. (2008). Tin Chemistry: Fundamentals, Frontiers, and Applications.

[4] Schäfer S.G., Femfert U. (1984). Tin—A toxic heavy metal? A review of the literature. Regul. Toxicol. Pharmacol..

[5] Schulz K.J., DeYoung J.H., Seal R.R., Bradley D.C. (2017). Critical Mineral Resources of the United States—Economic and Environmental Geology and Prospects for future Supply.

[6] Rüdel H. (2003). Case study: Bioavailability of tin and tin compounds. Ecotoxicol. Environ. Saf..

[7] Blunden S., Wallace T. (2003). Tin in canned food: A review and understanding of occurrence and effect. Food Chem. Toxicol..

[8] Ostrakhovitch E.A., Cherian M.G. (2007). Tin. Handbook on the Toxicology of Metals.

[9] Tuzen M., Uluozlu O.D., Mendil D., Soylak M., Machado L.O.R., dos Santos W.N.L., Ferreira S.L.C. (2018). A simple, rapid and green ultrasound assisted and ionic liquid dispersive microextraction procedure for the determination of tin in foods employing ETAAS. Food Chem..

[10] Wei X., Jang G., Roper D.K. (2015). Spectrophotometric determination of tin(II) by redox reaction using 3,3′,5,5′–tetramethylbenzidine dihydrochloride and N–bromosuccinimide. J. Anal. Chem..

[11] Olmedo P., Pla A., Hernández A.F., Barbier F., Ayouni L., Gil F. (2013). Determination of toxic elements (mercury, cadmium, lead, tin and arsenic) in fish and shellfish samples. Risk assessment for the consumers. Environ. Int..

[12] Khodadoust S., Cham Kouri N. (2014). Preconcentration of Sn (II) using the methylene blue on the activated carbon and its determination by spectrophotometry method. Spectrochim. Acta A Mol. Biomol. Spectrosc..

[13] Huang C., Li Q., Mo J., Wang Z. (2016). Ultratrace determination of tin, germanium, and selenium by hydride generation coupled with a novel solution-cathode glow discharge-atomic emission spectrometry method. Anal. Chem..

[14] Muniz L.P., dos Santos L.M.G., do Couto K.L.M., Jacob S. (2018). Evaluation of metals in tomato sauces stored in different types of packaging. Food Sci. Technol..

[15] Morte E.S.d.B., Barbosa I.d.S., Santos E.C., Nobrega J.A., Korn M.d.G.A. (2012). Axial view inductively coupled plasma optical emission spectrometry for monitoring tin concentration in canned tomato sauce samples. Food Chem..

[16] Mino Y. (2006). Determination of tin in canned foods by X-ray fluorescence spectrometry. J. Health Sci..

[17] Fritz J.S., Goodkin L. (1974). Separation and determination of tin by liquid-solid chromatography. Anal. Chem..

[18] Ye Y., Sang J., Ma H., Tao G. (2010). Gas-phase chemiluminescence with ozone oxidation for the determination of total tin in environmental samples using flow injection hydride generation and cryotrapping. Anal. Chim. Acta.

[19] Bard A.J., Faulkner L.R., White H.S. (2022). Electrochemical Methods: Fundamentals and Applications.

[20] Phillips S.L., Shain I. (1962). Application of stripping analysis to the trace determination of tin. Anal. Chem..

[21] Méndez J.H., Martínez R.C., López M.E.G. (1982). Simultaneous determination of tin and lead by a.c. anodic stripping voltammetry at a hanging mercury drop electrode sensitized by cetyltrimethylammoniu. Anal. Chim. Acta.

[22] Fano V., Zanotti L. (1973). Trace determination of tin(II) and tin(IV) in different solvents (H_2_O, CH_3_OH) by anodic stripping voltammetry. Microchem. J..

[23] Dirilgen N., Dogan F. (2004). Anodic stripping voltammetry: Sn and Pb analysis in archaeometallurgical samples. Int. J. Environ. Anal. Chem..

[24] Li Y.-H., Xie H.-Q., Zhou F.-Q., Guo H.-S. (2006). Determination of trace tin by anodic stripping voltammetry at a carbon paste electrode. Electroanalysis.

[25] Prior C., Walker G.S. (2006). The use of the bismuth film electrode for the anodic stripping voltammetric determination of tin. Electroanalysis.

[26] Hutton E.A., Hočevar S.B., Mauko L., Ogorevc B. (2006). Bismuth film electrode for anodic stripping voltammetric determination of tin. Anal. Chim. Acta.

[27] Prior C. (2010). Anodic stripping voltammetry of tin at the bismuth film electrode using cetyltrimethylammonium bromide. Electroanalysis.

[28] Frena M., Campestrini I., de Braga O.C., Spinelli A. (2011). In situ bismuth-film electrode for square-wave anodic stripping voltammetric determination of tin in biodiesel. Electrochim. Acta.

[29] Tyszczuk-Rotko K., Metelka R., Vytřas K., Barczak M., Sadok I., Mirosław B. (2016). A simple and easy way to enhance sensitivity of Sn(IV) on bismuth film electrodes with the use of a mediator. Monatsh. Für Chem. Chem. Mon..

[30] Yang G., Wang Y., Qi F. (2012). Differential pulse anodic stripping voltammetric determination of traces of tin using a glassy carbon electrode modified with bismuth and a film of poly(bromophenol blue). Microchim. Acta.

[31] Sobhanardakani S., Farmany A., Abbasi S. (2014). A new modified multiwalled carbon nanotube paste electrode for quantification of tin in fruit juice and bottled water samples. J. Ind. Eng. Chem..

[32] Faller C., Henze G., Stojko N., Saraeva S., Brainina K. (1997). Modified solid electrodes for stripping voltammetric determination of tin. Fresenius J. Anal. Chem..

[33] Yang S., Tian H., Wang D., Tang Y. (1995). The determination of trace tin by cathodic stripping voltammetry with a Nafion^®^-modified electrode. J. Electroanal. Chem..

[34] Wang J., Zadeii J. (1987). Ultrasensitive and selective measurements of tin by adsorptive stripping voltammetry of the tin—Tropolone complex. Talanta.

[35] van den Berg C.M.G., Khan S.H., Riley J.P. (1989). Determination of tin in sea water by adsorptive cathodic stripping voltammetry. Anal. Chim. Acta.

[36] Adeloju S.B. (1991). Adsorptive voltammetric stripping analysis of ultra-trace amounts of tin in natural waters and sediments. Anal. Sci..

[37] Li Y.-H., Long H., Zhou F.-Q. (2005). Determination of trace tin by catalytic adsorptive cathodic stripping voltammetry. Anal. Chim. Acta.

[38] Abbasi S., Hamdeghadareh S., Rezaei-Soufi L., Farmany A. (2020). 2,2-Hydroxyphenylbenzoxazole as a selective chelating agent for complexation with Tin and Zinc: A voltammetry study. Eurasian Chem. Commun..

[39] Gao Z., Siow K.S. (1996). Adsorptive stripping differential pulse voltammetric determination of trace amounts of tin in biological samples. Anal. Sci..

[40] Adeloju S.B.O., Pablo F. (1992). Determination of ultra-trace concentrations of tin by adsorptive cathodic stripping voltammetry on a glassy carbon mercury film electrode. Anal. Chim. Acta.

[41] Heppeler F., Sander S., Henze G. (1996). Determination of tin traces in water samples by adsorptive stripping voltammetry. Anal. Chim. Acta.

[42] Adamczyk M., Grabarczyk M. (2022). Application of a solid bismuth microelectrode in an adsorptive stripping voltammetric procedure of trace tin quantification. J. Electrochem. Soc..

[43] Grabarczyk M., Wlazłowska E., Wawruch A. (2023). Stripping voltammetry with nanomaterials-based electrode in the environmental analysis of trace concentrations of tin. ChemPhysChem.

[44] Glodowski S., Kublik Z. (1980). Cyclic and stripping voltammetry of tin at mercury hanging drop and film electrodes in acidic o-diphenol media in the presence of lead and cadmium. Anal. Chim. Acta.

[45] Dadda A.S., Teixeira A.C., Feltes P.K., Campos M.M., Leite C.E., Moriguchi-Jeckel C.M. (2014). Determination of Sn^2+^ in lyophilized radiopharmaceuticals by voltammetry, using hydrochloric acid as electrolyte. J. Braz. Chem. Soc..

[46] Adeloju S.B.O., Pablo F. (1995). Simultaneous determination of lead and tin in biological and environmental materials by differential pulse cathodic voltammetry on a glassy carbon mercury film electrode. Electroanalysis.

[47] McCrory-Joy C., Rosamilia J.M. (1982). Differential pulse polarography of germanium(IV), tin(IV), arsenic(V), antimony(V), selenium(IV) and tellurium(VI) at the static mercury drop electrode in catechol—Perchlorate media. Anal. Chim. Acta.

[48] Ni Y., Wang L. (1999). Simultaneous polarographic determination of lead(II) and tin(II) by multivariate calibration. Anal. Lett..

[49] Hubert C., Ziémons E., Rozet E., Breuer A., Lambert A., Jasselette C., de Bleye C., Lejeune R., Hubert P. (2010). Development and validation of a quantitative method for the selective determination of tin species in tin octoate by differential pulse polarography. Talanta.

[50] Weber G. (1986). Determination of tin in the ng g^−1^ range differential pulse polarography. Anal. Chim. Acta.

[51] Qiong L., Guanghan L., Heng W., Xiaogang W. (1999). Determination of trace tin in foods by single-sweep polarography. Food Chem..

[52] Somer G., Ünal Ü. (2011). Simultaneous determination of trace Sn(II) and Sn(IV) using differential pulse polarography and application. Turk. J. Chem..

[53] Somer G., Arslantas A. (1994). Simultaneous determination of tin, lead and molybdenum by differential-pulse polarography. Analyst.

[54] Decristoforo C., Obendorf D., Reichart E., Stubauer G., Riccabona G. (1998). Determination of Sn(II) in technetium cold kits by voltammetry at the hanging mercury drop electrode (HMDE) and relevant radiopharmaceutical applications. Nucl. Med. Biol..

[55] Pérez-Herranz V., García-Gabaldón M., Guiñón J., García-Antón J. (2003). Effect of citric acid and hydrochloric acid on the polarographic behaviour of tin. Anal. Chim. Acta.

[56] Taher M.A., Puri B.K. (1999). Differential pulse polarographic determination of tin in alloys and environmental samples after preconcentration with the ion pair of 2-nitroso-1-naphthol-4-sulfonic acid and tetradecyldimethylbenzylammonium chloride onto microcrystalline naphthalene or by column method. Talanta.

[57] Monticelli D., Psaro R., Pozzi A., Dossi C., Recchia S. (2005). Differential pulse voltammetric determination of tin in the presence of noble metals. Anal. Bioanal. Chem..

[58] Hosseini M., Bagheri Sadeghi H., Rahimi M., Salavati-Niasari M., Dehghan Abkenar S., Alizadeh K., Reza Ganjali M. (2009). Highly selective and sensitive tin(II) membrane electrode based on a new synthesized schiff’s base. Electroanalysis.

[59] Aghaie H., Giahi M., Monajjemi M., Arvand M., Nafissi G.H., Aghaie M. (2005). Tin(II)-selective membrane potentiometric sensor using a crown ether as neutral carrier. Sens. Actuators B Chem..

[60] Arvand M., Moghimi A.M., Afshari A., Mahmoodi N. (2006). Potentiometric membrane sensor based on 6-(4-nitrophenyl)-2,4-diphenyl-3,5-diaza-bicyclo[3.1.0]hex-2-ene for detection of Sn(II) in real samples. Anal. Chim. Acta.

[61] Demkin A.M. (2006). Determination of trace tin by stripping potentiostatic coulometry. J. Anal. Chem..

[62] Bard A.J., Faulkner L.R. (2001). Fundamentals and applications. Electrochem. Methods.

[63] Borrill A.J., Reily N.E., Macpherson J.V. (2019). Addressing the practicalities of anodic stripping voltammetry for heavy metal detection: A tutorial review. Analyst.

[64] Sharma S., Mehtab S., Zaidi M.G. (2020). Voltammetry: An Electrochemical Analytical Method. Chem. Sci..

[65] Heyrovský M. (2011). Polarography—Past, present, and future. J. Solid State Electrochem..

[66] Gajdar J., Horakova E., Barek J., Fischer J., Vyskocil V. (2016). Recent applications of mercury electrodes for monitoring of pesticides: A critical review. Electroanalysis.

[67] Bobacka J., Ivaska A., Lewenstam A. (2008). Potentiometric ion sensors. Chem. Rev..

[68] Ding J., Qin W. (2020). Recent advances in potentiometric biosensors. TrAC Trends Anal. Chem..

[69] Shao Y., Ying Y., Ping J. (2020). Recent advances in solid–contact ion–selective electrodes: Functional materials, transduction mechanisms, and development trends. Chem. Soc. Rev..

[70] Blair E.O., Corrigan D.K. (2019). A review of microfabricated electrochemical biosensors for DNA detection. Biosens. Bioelectron..

[71] Porada R., Jedlińska K., Lipińska J., Baś B. (2020). Review–voltammetric sensors with laterally placed working electrodes: A review. J. Electrochem. Soc..

